# Large language model ensemble for automated TNM staging from radiology reports

**DOI:** 10.1093/bioinformatics/btag399

**Published:** 2026-07-14

**Authors:** Wen-Chao Yeh, Yi-Shin Chen, Wen-Lian Hsu, Shuntaro Yada, Yung-Chun Chang

**Affiliations:** Institute of Information Systems and Applications, National Tsing Hua University, Hsinchu City, 300, Taiwan; Department of Computer Science and Engineering, National Chung Hsing University, Taichung City, 402, Taiwan; Institute of Information Systems and Applications, National Tsing Hua University, Hsinchu City, 300, Taiwan; Institute of Information Systems and Applications, National Tsing Hua University, Hsinchu City, 300, Taiwan; Department of Computer Science and Information Engineering, Asia University, Taichung City, 413, Taiwan; Institute of Library, Information and Media Science, University of Tsukuba, Tsukuba, 305-8550, Japan; Graduate Institute of Data Science, Taipei Medical University, New Taipei City, 235, Taiwan; Clinical Big Data Research Center, Taipei Medical University Hospital, Taipei City, 110, Taiwan

## Abstract

**Motivation:**

Accurate TNM staging from lung cancer radiology reports is crucial for treatment planning and prognosis assessment. Manual staging processes are time-consuming and subject to inter-observer variability. Large language models (LLMs) offer opportunities to automate TNM staging with enhanced interpretability and clinical reasoning.

**Results:**

We developed two complementary systems for automated TNM staging from English radiology reports. System I employs GPT-4o with reasoning-based few-shot learning and multi-step voting. System II integrates multiple LLMs (GPT-4o and Gemini-2) using DSPy framework with MIPROv2 optimization. In NTCIR-18 RadNLP 2024 English main task, our approaches achieved first (joint accuracy: 0.6543) and second place (joint accuracy: 0.6296), demonstrating superior performance in T, N, and M classification with accuracies of 0.7037/0.9136/0.8889 and 0.7284/0.9383/0.8395, respectively.

**Availability and Implementation:**

Source code freely available at https://github.com/nlptmu/multi-expert-tnm-staging under MIT license. An archival snapshot of the version used in this study is deposited on Zenodo at https://doi.org/10.5281/zenodo.20338561. Implemented in Python 3.12+ with PyTorch 2.6 and DSPY 3.0, supporting Linux.

## 1 Introduction

Lung cancer is a leading cause of cancer death worldwide, making early diagnosis and accurate staging crucial for patient prognosis (Sung *et al.* 2021). The TNM (Tumor-Node-Metastasis) staging system serves as the international standard for assessing lung cancer progression and directly influences treatment planning and survival rates (Detterbeck *et al.* 2017, Wang *et al.* 2019). Chest CT radiology reports provide essential TNM-related information in narrative form, describing primary tumor characteristics, lymph node involvement, and distant metastases (Nobel *et al.* 2021, Puts *et al.* 2023). However, this clinically valuable information exists in unstructured free-text format, making manual extraction time-consuming, error-prone, and subject to inter-observer variability (Hassanpour *et al.* 2017, Liu *et al.* 2020).

Medical NLP for cancer staging has evolved significantly over two decades. Early approaches combined rule-based systems with traditional machine learning, using expert-crafted rules and statistical classifiers to extract staging information from pathology and radiology reports (McCowan *et al.* 2006, Wang *et al.* 2018). While these methods established feasibility, they required extensive feature engineering and struggled with complex linguistic phenomena such as uncertainty and temporality (Zhang *et al.* 2016, Lou *et al.* 2019). Deep learning subsequently introduced distributed representations and neural networks that reduced reliance on hand-crafted features (Yang *et al.* 2018, Spandorfer *et al.* 2019, Lee *et al.* 2020), but these models typically required large labeled datasets and offered limited interpretability (Sorin *et al.* 2020, Montazeri *et al.* 2021).

Large language models (LLMs) represent a paradigm shift in clinical NLP, offering zero-/few-shot learning capabilities through prompt engineering and demonstrating strong performance in clinical information extraction without task-specific fine-tuning (Sivarajkumar *et al.* 2024). In pulmonary oncology, existing TNM extraction systems have shown promise (Nobel *et al.* 2021, Puts *et al.* 2023), but largely rely on conventional models without fully exploiting LLMs’ reasoning capabilities or zero-/few-shot generalization. Meanwhile, ensemble learning has proven effective in medical AI for improving accuracy and robustness (Kim and Meystre 2019, Yang *et al.* 2025), yet its application to LLMs for complex staging tasks remains underexplored.

This study addresses these gaps by proposing the first large language model ensemble framework for automated lung cancer TNM staging from radiology reports, evaluated on the NTCIR-18 RadNLP 2024 shared task (Nakamura *et al.* 2025). We developed two complementary systems: System I (Expert-enhanced few-shot ensemble) employs GPT-4-based clinical reasoning with expert-grounded few-shot prompting, while System II (Multi-model Expert Panel) implements a multi-expert LLM ensemble using automated prompt optimization. Our approaches achieved first (joint accuracy: 0.6543) and second place (joint accuracy: 0.6296) in the English main task, demonstrating top-ranked performance in fine-grained T/N/M classification with accuracies of 0.7037/0.9136/0.8889 and 0.7284/0.9383/0.8395, respectively. This work makes three key contributions: (i) first demonstration that LLM ensembles with explicit reasoning achieve best performance in the NTCIR-18 RadNLP 2024 English main task on fine-grained TNM staging from real-world radiology reports, (ii) systematic analysis of how different LLM experts and voting strategies affect staging decisions, and (iii) empirical evidence for LLM-based multi-expert decision support in clinically realistic evaluation settings.

## 2 Literature review

### 2.1 TNM staging and clinical NLP evolution

Lung cancer remains the leading cause of cancer death globally (Sung *et al.* 2021), with the TNM system serving as the cornerstone for treatment selection and prognostic stratification (Brierley *et al.* 2016, J. L. C. Society 2021). Chest CT radiology reports provide essential TNM information but exist as unstructured narratives with heterogeneous templates and complex linguistic phenomena such as hedging, negation, and temporal qualifiers (Pons *et al.* 2016, Hassanpour *et al.* 2017). While NLP methods can structure radiology text and infer oncologic outcomes, only a small number of systems have undergone robust clinical validation (Montazeri *et al.* 2021).

Early medical NLP systems relied on rule-based approaches and traditional machine learning. Systems like MedLEE mapped radiology reports to structured concepts but were constrained by vocabulary coverage and rule completeness (Liu *et al.* 2019). Statistical classifiers combined with tokenization, negation detection, and n-gram features showed feasibility for cancer staging (McCowan *et al.* 2006, Zhang *et al.* 2016, Wang *et al.* 2018), but these approaches were heavily dependent on manually selected features and struggled with complex linguistic phenomena (Zeng *et al.* 2018, Lou *et al.* 2019).

Deep learning subsequently alleviated these limitations by learning hierarchical representations directly from text. Word embeddings, CNNs, and RNNs were increasingly applied to radiology NLP (Spandorfer *et al.* 2019, Lee *et al.* 2020), with neural approaches outperforming traditional methods across many tasks (Sorin *et al.* 2020). However, relatively few deep learning systems have targeted full TNM staging, particularly with fine-grained subcategories (Montazeri *et al.* 2021, Puts *et al.* 2023).

### 2.2 Large language models and ensemble methods

Large language models have transformed clinical NLP by enabling flexible zero-/few-shot learning via prompting rather than task-specific training (Sivarajkumar *et al.* 2024). Systematic evaluation of prompting strategies—including chain-of-thought, heuristic, and ensemble prompts—has shown substantial performance improvements in clinical tasks, though optimal strategies vary by task and model (Sivarajkumar *et al.* 2024). In pulmonary oncology, existing TNM extraction systems have demonstrated feasibility (Nobel *et al.* 2021, Puts *et al.* 2023), but most predate modern LLMs or only partially exploit their reasoning capabilities.

Ensemble learning has proven effective in medical AI by combining multiple models to improve accuracy and robustness (Kim and Meystre 2019). These approaches reduce bias and variance while providing closer analogies to multi-expert decision-making in clinical teams. However, systematic exploration of LLM ensembles for clinical text tasks such as TNM staging remains limited, representing a significant gap in current research.

### 2.3 NTCIR RadNLP context

The NTCIR-17 MedNLP-SC shared task established automated cancer staging from radiology reports as a benchmark challenge, achieving accuracies of 67%, 80%, and 93% for T, N, and M categories respectively on Japanese reports (Nakamura *et al.* 2023). Participating teams demonstrated diverse approaches: Team KRad leveraged GPT-3.5-turbo through zero-shot prompting (Nishio *et al.* 2023), Team kuhp fine-tuned Japanese language models with data augmentation (Fujimoto *et al.* 2023), and Team NAIST-SOCRR employed bidirectional transformer models (Fukushima *et al.* 2023). These approaches highlighted the importance of leveraging both representation and generative models, sophisticated prompt engineering, and domain expertise integration.

The evolution from NTCIR-17 to NTCIR-18 RadNLP has illuminated key strategies for automated cancer staging, yet systematic evidence regarding LLM ensemble design for fine-grained TNM staging under real-world clinical constraints remains limited. Our study addresses this gap by demonstrating that multi-expert LLM ensembles with explicit reasoning and prompt optimization can achieve top-ranked performance on fine-grained lung cancer TNM staging from real-world radiology reports in a multilingual setting.

## 3 Materials and methods

Our research team participated in the NTCIR-18 RadNLP Lung Cancer TNM Staging main task (Nakamura *et al.* 2025), which represents a substantial evolution from the previous NTCIR-17 RR-TNM framework (Nakamura *et al.* 2023). The NTCIR-18 RadNLP 2024 task introduces enhanced classification granularity that better reflects clinical staging requirements in real-world oncology practice. The task expanded from a simplified 3-label, 2–5 class system to a comprehensive 3-label, 4–10 class classification framework, incorporating clinically relevant suffix-based categories. This enhanced granularity aligns with the 8th edition of the TNM Classification of Malignant Tumours, providing a more precise automated staging system that can support clinical decision-making processes.

- **T categories**: T0, Tis, T1mi, T1a, T1b, T1c, T2a, T2b, T3, T4
- **N categories**: N0, N1, N2, N3
- **M categories**: M0, M1a, M1b, M1c

### 3.1 Datasets

The CT images and case presentations were sourced from Radiopaedia, an open-access radiology reference platform. The radiology reports were not taken from Radiopaedia; rather, nine board-certified radiologists recruited for this task independently wrote free-text reports in Japanese for each case, based on their review of the Radiopaedia CT images. TNM stage labels were independently assigned by two separate board-certified radiologists following the 8th edition criteria of the Japan Lung Cancer Society (JLCS), with any discrepancies resolved by consensus.

The original Japanese corpus was systematically constructed using lung cancer cases sourced from Radiopaedia, an open-access radiology reference platform. The case selection followed a rigorous five-step protocol conducted by a board-certified radiologist with six years of diagnostic radiology experience. This process included initial keyword screening, rule-based filtering to remove cases lacking patient demographics, brief manual review of case titles and CT images to exclude non-primary lung cancer cases, detailed examination to eliminate highly equivocal findings, and final manual selection to ensure diverse representation of cancer staging findings (Nakamura *et al.* 2025). The corpus comprises 378 radiology reports from 42 lung cancer cases, with 27 cases (243 reports) created for NTCIR-17 (Nakamura *et al.* 2023) and 15 cases (135 reports) originally developed for NTCIR-16 (Nakamura *et al.* 2022, Yada *et al.* 2022).

Report generation involved nine board-certified radiologists. Each radiologist independently created free-text Japanese radiology reports that included hyperlinks to Radiopaedia cases, patient demographics, and case presentations. To maintain realistic clinical conditions, no explicit reporting instructions were provided, and radiologists worked independently without discussion, with fictitious patient information included to help resolve equivocal imaging findings. The dataset was split at the case level, ensuring that all reports derived from the same clinical case were assigned exclusively to one partition. The English track comprises 12 training cases (108 reports), 6 validation cases (54 reports), and 9 test cases (81 reports), with no case appearing in more than one subset. This case-level design prevents leakage of reports derived from the same clinical case across training, validation, and test partitions.

**Figure 1:**
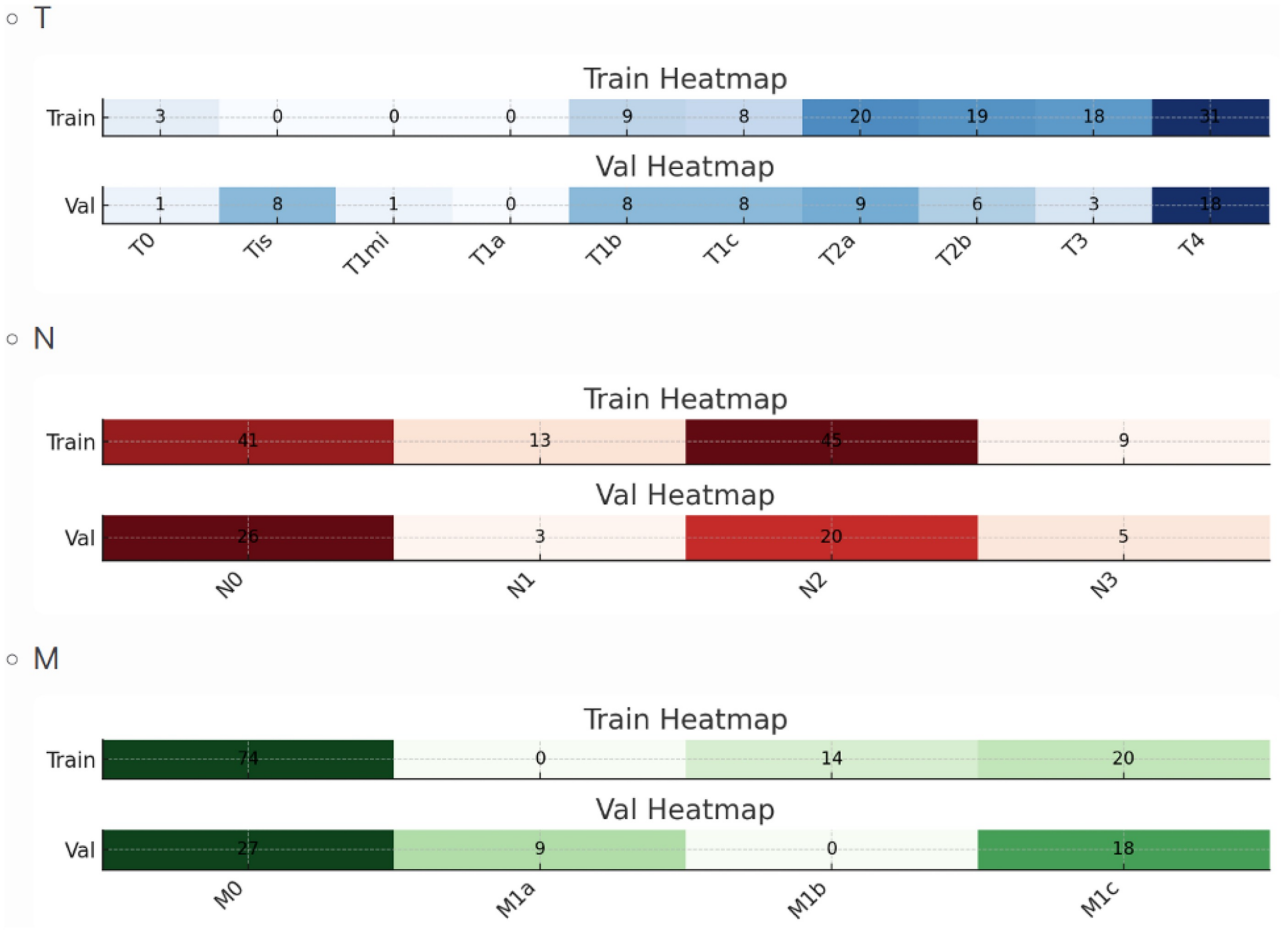
Heatmap representation of TNM classification distribution in training and validation data.

The training and validation datasets exhibit significant class imbalances that present methodological challenges for model development and evaluation. T classification demonstrates pronounced imbalance, with T2b (training: 20, validation: 9) and T4 (training: 31, validation: 18) representing the most frequent categories, while early-stage classifications such as T1mi and T1a remain severely underrepresented. N classification shows similar patterns, with N0 (training: 41, validation: 26) and N2 (training: 45, validation: 20) dominating the distribution, whereas N3 cases appear infrequently across both datasets. The most extreme imbalance occurs in M classification, where M0 cases (training: 74, validation: 27) vastly outnumber metastatic subcategories, particularly M1a (training: 0, validation: 9) and M1b (training: 14, validation: 0). These distribution patterns create substantial challenges for model generalization, especially for minority classes that may be clinically significant but statistically underrepresented, potentially affecting the system’s ability to accurately identify rare but important staging categories in clinical applications. A heatmap respresstation of TNM dataset is illustrated in Fig. 1.

### 3.2 Clinical validation and annotation philosophy analysis

Clinical validation of the training dataset revealed fundamental differences between annotation methodology and real-world clinical decision-making processes. Two experienced clinicians from our team—a thoracic oncology specialist with over 10 years of experience and a pulmonologist with nearly 30 years of clinical practice—conducted comprehensive review of the training annotations. Their analysis identified a systematic pattern where dataset annotations adopted more conservative interpretations compared to standard clinical practice, creating potential misalignment between model training objectives and clinical reasoning expectations.

Case study analysis demonstrates these philosophical differences through radiology report “1 679 413,” which presents ambiguous findings requiring clinical judgment.

Emphysematous changes.

A 47 mm irregular mass in the left upper lobe, suspicious of lung cancer. Possible invasion into the left pulmonary artery, as far as can be assessed with CT to a limited extent. The mass is also close to the aortic arch.

A small nodule is present in the right middle lobe, possibly inflammatory or metastasis, re-evaluation recommended in follow-up.

No significant mediastinal lymph node enlargement.

No pleural effusion.

Clinical experts interpreted *“possible invasion into the left pulmonary artery”* as sufficient evidence for T4 classification despite imaging limitations, while annotators assigned T2b based solely on tumor size due to uncertainty language. Similarly, for M classification, clinicians classified the contralateral nodule as M1a based on metastatic possibility, whereas annotators maintained M0 pending definitive follow-up evaluation. These systematic differences highlight the inherent subjectivity in TNM staging interpretation and underscore the necessity for explicit reasoning mechanisms in automated classification systems.

### 3.3 System I: expert-enhanced few-shot ensemble architecture

System I addresses these clinical validation findings through a three-component architecture that incorporates structured reasoning, contextual learning, and consensus mechanisms. The system employs GPT-4o (OpenAI 2024) to generate structured inference rationales for each training case by analyzing pathology reports, TNM labels, and staging guidelines to reconstruct annotation logic. These rationales serve as contextual references that capture nuanced decision-making processes not explicitly encoded in original labels, creating a knowledge base that bridges the gap between conservative annotations and clinical reasoning patterns.

**Figure 2:**
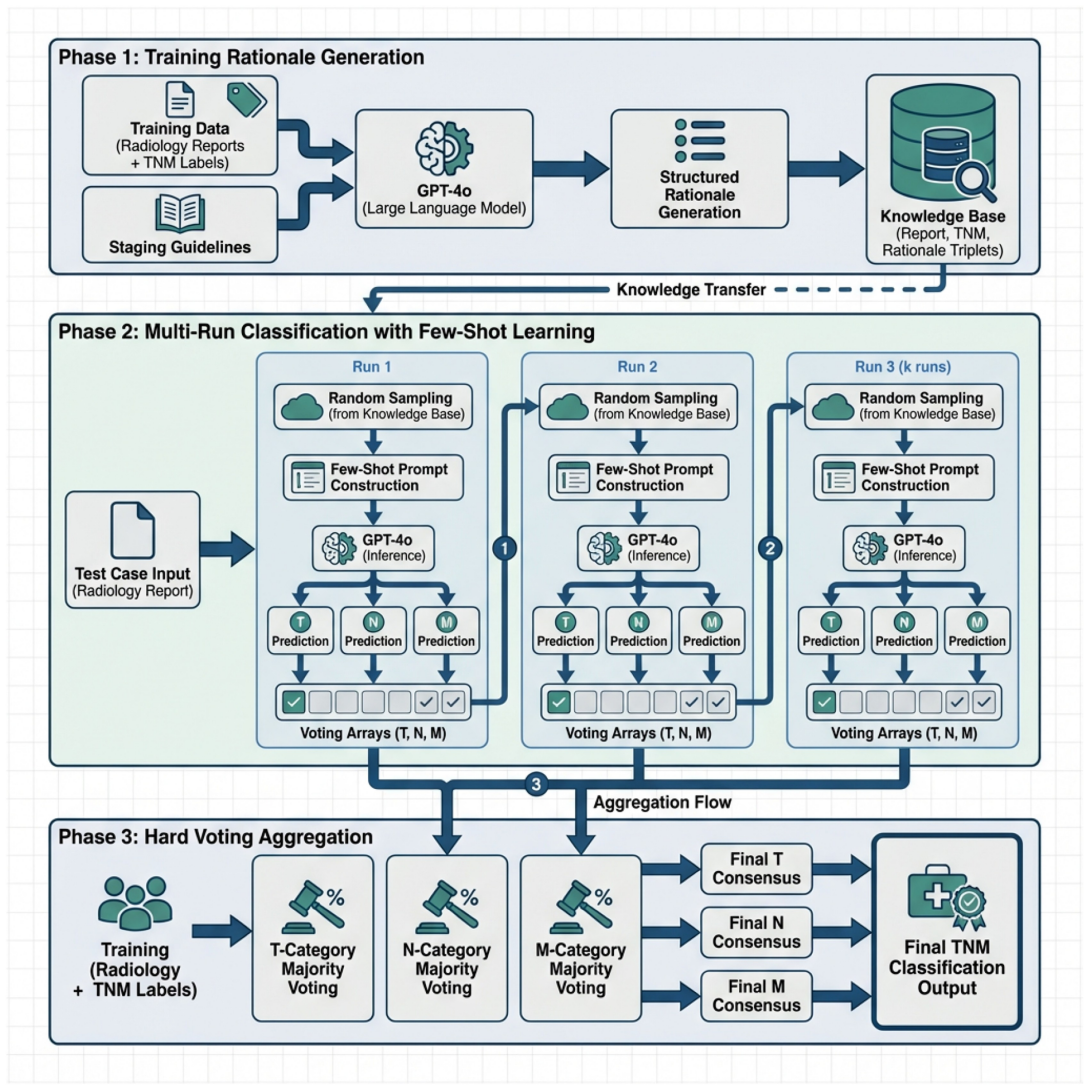
System I Expert-enhanced few-shot ensemble Architecture.

The classification process implements few-shot learning methodology where each test case incorporates randomly selected training samples—including pathology reports, TNM labels, and generated reasoning—within the prompt context. This approach grounds GPT-4o predictions in established annotation patterns while maintaining consistency with dataset philosophy. To enhance reliability and mitigate stochastic variability, the system employs a hard-voting mechanism across multiple independent inference runs, each utilizing different randomly sampled few-shot examples. The predicted T, N, and M categories from these runs undergo majority voting aggregation, improving classification robustness and reducing inconsistencies inherent in single-run predictions, as illustrated in Fig. 2 and Algorithm 1. The values of *k* and *n* were determined through a systematic parameter search on the validation set, evaluating combinations of k∈{1,3,5} and n∈{3,5,7,9}. The combination of k=3 and n=7 yielded the best joint accuracy on the validation set and was therefore selected as the final configuration. Increasing *k* from 1 to 3 enhanced validation performance by mitigating stochastic variability across independent inference runs; however, no marginal gains were observed for k>3. For few-shot learning, n=7 examples optimized the trade-off between contextual guidance and prompt length. While this configuration increased exposure to diverse annotation patterns, it remains inherently limited by the training set’s distribution and cannot account for absent categories. However, it did not and could not guarantee coverage of categories absent from the training set, such as M1a. Random sampling was chosen over similarity-based retrieval after empirical comparison on the validation set. Similarity-based retrieval—selecting the most semantically similar training cases for each test input—is a common strategy in few-shot learning. However, given the relatively small size of the dataset and the high homogeneity of the clinical cases (most reports share similar vocabulary and staging patterns), similarity-based retrieval consistently returned a narrow, overlapping set of examples. This reduced the diversity of the few-shot context and led to lower validation performance compared to random sampling. Random sampling, combined with the multi-run voting mechanism, introduced diversity across inference runs and provided broader exposure to available annotation patterns, although it could not compensate for absent or extremely rare categories.

**Figure 3:**
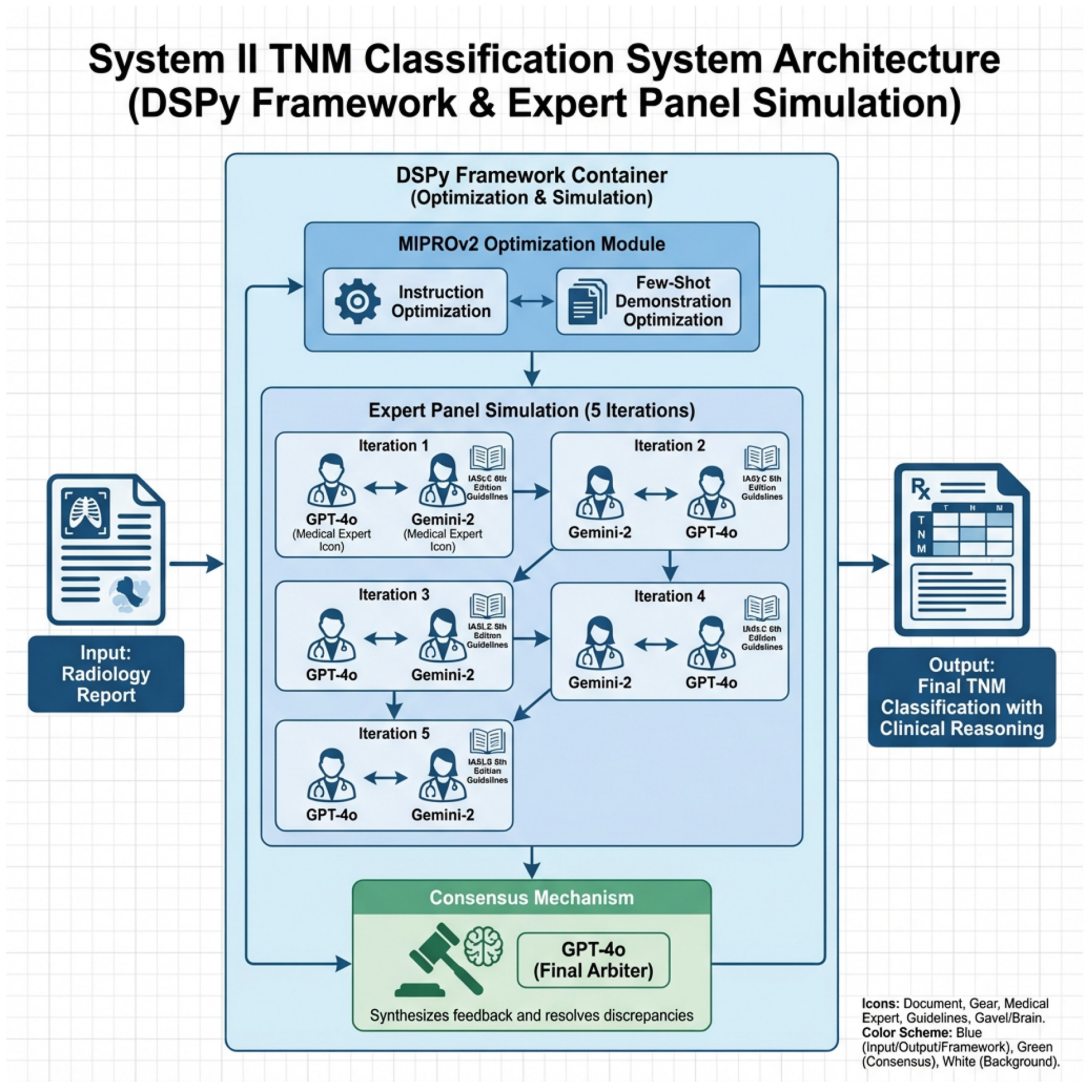
System II Multi-model Expert Panel Architecture.

**Figure 4:**
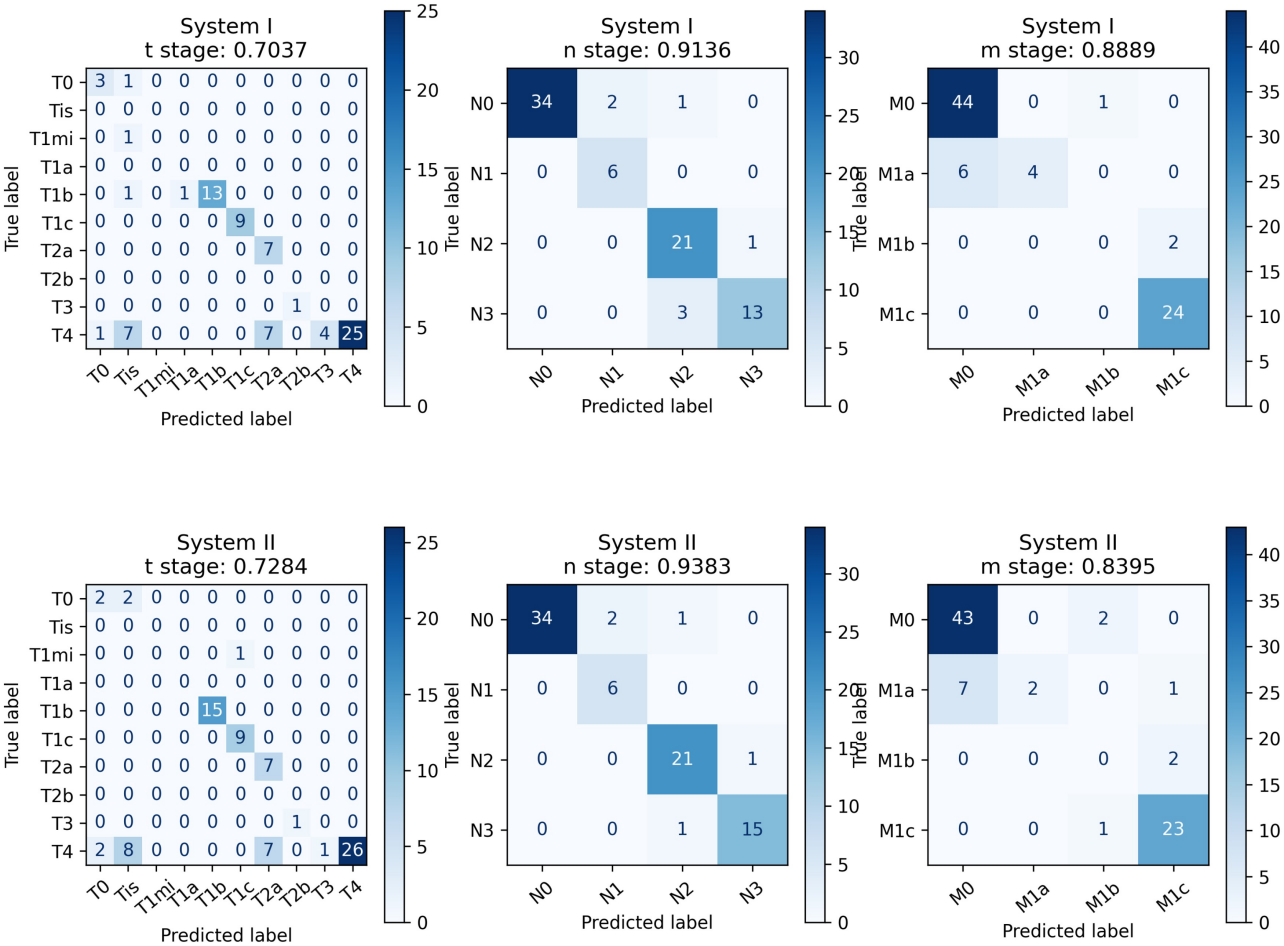
Confusion matrices for T, N, and M classification on the test set (n=81) for System I (top) and System II (bottom). Each panel shows the distribution of predicted versus true labels for fine-grained TNM subcategories. Rare categories with zero training instances (M1a) or zero validation instances (M1b) show the lowest per-class accuracy, confirming that aggregate accuracy metrics mask critical performance gaps in underrepresented classes.

Algorithm 1 System I for TNM Classification with Structured Reasoning

**Require:** Training data $D_{\mathrm{train}}$, Test case $x_{\mathrm{test}}$, Number of voting runs *k* (set to 3), Number of few-shot examples *n* (set to 7)

**Ensure:** Final TNM classification $\left(T_{\mathrm{final}},N_{\mathrm{final}},M_{\mathrm{final}}\right)$

1: **Phase 1: Training Rationale Generation** 

2: **for** each case $(\mathrm{repor}t_{i},TNM_{i})\in D_{\mathrm{train}}$  **do** 

3: $\mathrm{rational}e_{i}\leftarrow$ GPT-4o$\left(\mathrm{repor}t_{i},TNM_{i},\mathrm{staging}\_\mathrm{guidelines}\right)$

4:  Store $\left(\mathrm{repor}t_{i},TNM_{i},\mathrm{rational}e_{i}\right)$ in knowledge base

5: **end for** 

6:

7: **Phase 2: Multi-Run Classification with Few-Shot Learning** 

8: Initialize voting arrays: $T_{\mathrm{votes}}[],N_{\mathrm{votes}}[],M_{\mathrm{votes}}[]$

9: **for**  run=1 to *k* **do** 

10:  few_shot_samples← RandomSample$\left(D_{\mathrm{train}},n\right)$

11:  prompt← ConstructPrompt$\left(x_{\mathrm{test}},\mathrm{few}\_\mathrm{shot}\_\mathrm{samples}\right)$

12:  $(T_{\mathrm{pred}},N_{\mathrm{pred}},M_{\mathrm{pred}})\leftarrow$ GPT-4o(prompt)

13:  $T_{\mathrm{votes}}$.append$\left(T_{\mathrm{pred}}\right)$

14:  $N_{\mathrm{votes}}$.append$\left(N_{\mathrm{pred}}\right)$

15:  $M_{\mathrm{votes}}$.append$\left(M_{\mathrm{pred}}\right)$

16: **end for** 

17:

18: **Phase 3: Hard Voting Aggregation** 

19: $T_{\mathrm{final}}\leftarrow$ MajorityVote$\left(T_{\mathrm{votes}}\right)$

20: $N_{\mathrm{final}}\leftarrow$ MajorityVote$\left(N_{\mathrm{votes}}\right)$

21: $M_{\mathrm{final}}\leftarrow$ MajorityVote$\left(M_{\mathrm{votes}}\right)$

22:

23: **return**  $\left(T_{\mathrm{final}},N_{\mathrm{final}},M_{\mathrm{final}}\right)$

### 3.4 System II: multi-model expert panel architecture

System II implements an advanced multi-model consensus approach that simulates a collaborative medical expert panel for TNM staging classification (refer to Fig. 3 and Algorithm 2). The system leverages the DSPy framework (Khattab *et al.* 2022, 2024), which provides a declarative programming paradigm for building modular AI systems rather than relying on brittle prompt engineering. DSPy enables the construction of compositional Python code that teaches language models to deliver high-quality outputs through systematic optimization algorithms. This framework is particularly advantageous for medical applications because it allows for transparent, controllable, and adaptable AI solutions that can be systematically improved and validated.

The core architecture employs an alternating dual-model strategy using GPT-4o and Gemini-2, where each model functions as an independent medical expert analyzing radiology reports. This design mimics real-world clinical practice where multiple specialists review complex cases to reach consensus. Each language model operates under carefully crafted prompts that embody the expertise of skilled radiologists and oncologists, strictly adhering to the IASLC 8th edition lung cancer staging guidelines. The alternating pattern ensures diverse analytical perspectives while maintaining clinical consistency across evaluations.

The system’s performance is enhanced through MIPROv2 optimization (Opsahl-Ong *et al.* 2024), a sophisticated prompt optimizer that jointly optimizes both instructions and few-shot demonstrations. MIPROv2 employs Bayesian optimization to efficiently navigate the large search space of instruction-demonstration combinations, making it particularly effective for multi-stage language model programs. The optimizer works by bootstrapping few-shot example candidates, proposing instructions grounded in different task dynamics, and finding optimal combinations through systematic search.

This optimization approach is crucial for medical NLP tasks because it addresses the challenge of credit assignment across multiple modules while maximizing downstream performance metrics. MIPROv2’s demo-awareness capability allows it to understand which few-shot example pairs boost performance, enabling the system to learn from labeled examples and improve its clinical reasoning capabilities. The optimization process factorizes the problem into optimizing free-form instructions and few-shot demonstrations for every module, introducing strategies to craft task-grounded instructions that reflect clinical decision-making patterns.

Algorithm 2 System II: Multi-Model Expert Panel TNM Classification

**Require:** Radiology report *x*, Number of iterations k=5, Models {GPT4o,Gemini2}

**Ensure:** Final TNM classification $\left(T_{\mathrm{final}},N_{\mathrm{final}},M_{\mathrm{final}}\right)$ with clinical reasoning

1: **Phase 1: MIPROv2 Optimization** 

2: $\mathrm{instruction}s_{\mathrm{opt}}\leftarrow$

3:  MIPROv2.OptimizeInstructions(task_dynamics)

4: $\mathrm{demonstration}s_{\mathrm{opt}}\leftarrow$

5:  MIPROv2.OptimizeFewShot(labeled_examples)

6: prompts←

7:  $\mathrm{DSPy}.\mathrm{ComposePrompts}(\mathrm{instruction}s_{\mathrm{opt}},\mathrm{demonstration}s_{\mathrm{opt}})$

8: **Phase 2: Expert Panel Simulation** 

9: Initialize assessment arrays: assessments←[]

10: Set deterministic seeds for reproducibility

11: **for**  iteration=1 to *k* **do** 

12:  **if** *iteration* is odd **then** 

13:   current_model←GPT4o

14:  **else** 

15:   current_model←Gemini2

16:  **end if** 

17:  expert_prompt←

18:  ConstructExpertPrompt(prompts,IASLC_guidelines)

19:  $\mathrm{assessmen}t_{i}\leftarrow \mathrm{current}\_\mathrm{model}(x,\mathrm{expert}\_\mathrm{prompt})$

20:  *assessments*.append$\left(\mathrm{assessmen}t_{i}\right)$

21: **end for** 

22: **Phase 3: Consensus Synthesis** 

23: consensus_prompt←

24:  ConstructConsensusPrompt(assessments,

25:  IASLC_guidelines)

26: $(T_{\mathrm{final}},N_{\mathrm{final}},M_{\mathrm{final}},\mathrm{reasoning})\leftarrow$

27:  GPT4o(consensus_prompt)

28: **Phase 4: Quality Assurance** 

29: Validate output format and clinical consistency

30: Apply error handling and standardization protocols

31: **return**  $\left(T_{\mathrm{final}},N_{\mathrm{final}},M_{\mathrm{final}},\mathrm{reasoning}\right)$

The classification process operates through a five-iteration consensus mechanism where each radiology report undergoes multiple independent assessments. During each iteration, the system alternates between GPT-4o and Gemini-2 models, collecting diverse analytical opinions that capture different aspects of clinical reasoning. After completing all iterations, GPT-4o serves as the final arbiter, synthesizing the collected assessments into definitive TNM classifications accompanied by clinical reasoning justifications.

To ensure reproducibility and consistency, the implementation incorporates comprehensive error handling mechanisms, deterministic behavior through seed setting, and standardized data preprocessing protocols. The system maintains strict adherence to clinical guidelines while leveraging the modular programming advantages of DSPy, which allows for transparent optimization and systematic performance improvements. This technical framework enables the system to produce reliable TNM classifications that can be validated against clinical standards while maintaining the flexibility to adapt to evolving medical knowledge and staging criteria.

## 4 Experiments

Systems were evaluated under the NTCIR-18 RadNLP 2024 shared task (Table 1 shows the dataset quantities) framework using joint fine accuracy as the primary metric. The following sections present detailed analysis of the configuration parameters, optimization strategies, and performance metrics that characterized the experimental evaluation of our proposed TNM classification systems. The performance metrics of our various systems and another participations in the main task are presented in Table 2, respectively.

**Table 1:** NTCIR-18 RadNLP TNM staging task dataset quantities.

Dataset	English Track
Train	108
Validation	54
Test	81

**Table 2:** Model performance comparison: fine vs coarse accuracy metrics RadNLP 2024—The result of main task.

Rank	ID of participation	Fine				Coarse			
		Joint*	T^†^	N^‡^	M^§^	Joint**	T^††^	N^‡‡^	M^§§^
		accuracy	accuracy	accuracy	accuracy	accuracy	accuracy	accuracy	accuracy
1	Our System I	**0.6543** (±0.104)	0.7037 (±0.099)	0.9136 (±0.061)	0.8889 (±0.068)	0.6914	0.7407	0.9136	0.9136
2	Our system II	0.6296 (±0.105)	**0.7284** (±0.097)	0.9383 (±0.052)	0.8395 (±0.080)	0.6667	0.7407	0.9383	0.8889
3	CYUT	0.6049	0.6914	0.9383	**0.9259**	0.6296	0.7037	0.9383	**0.9383**
4	NLI24	0.5679	0.6914	**0.9506**	0.8395	**0.7160**	**0.8272**	**0.9506**	0.8519
5	tsukurad	0.5556	0.6049	**0.9506**	0.8765	0.6914	0.716	**0.9506**	0.9136
6	Our System III	0.5556	0.642	0.8889	0.8395	0.5802	0.6543	0.8889	0.8889
7	Our System IV	0.5309	0.6543	0.9136	0.8519	0.5802	0.6667	0.9136	0.9259
8	SINAI	0.5309	0.5926	0.9136	0.8889	0.5926	0.642	0.9136	0.9259
9	Hirosaki	0.5185	0.6543	0.9259	0.8395	0.5432	0.6667	0.9259	0.8642
10	Sociocom	0.4198	0.6296	0.7531	0.8272	0.5062	0.7037	0.7531	0.8642
11	NITKC team	0.2963	0.4568	0.8642	0.7778	0.4815	0.642	0.8642	0.8148
12	BSC-WN-MT	0.2963	0.4691	0.8272	0.7284	0.4938	0.642	0.8272	0.8765
13	RRR	0.2840	0.4815	0.7531	0.7654	0.4815	0.6296	0.7531	0.9012
14	UoM	0.1235	0.3333	0.5926	0.6914	0.2593	0.4444	0.5926	0.7901
15	RadAnalyzers	0	0.1111	0.6049	0.6543	0.0494	0.1358	0.6049	0.7654
16	RadAnalyzers	0	0	0	0	0	0	0	0

**Bold** indicates the best result in each column; underline indicates the second-best result in each column.

Note: Joint accuracy (fine)* is used to sort the leaderboard.

* Joint accuracy (fine)—The proportion of radiology reports with accurate predictions for all the T, N, and M factors.

† T accuracy (fine)—The proportion of radiology reports with accurate predictions for the T factor.

‡ N accuracy (fine)—The proportion of radiology reports with accurate predictions for the N factor.

§ M accuracy (fine)—The proportion of radiology reports with accurate predictions for the M factor.

** Joint accuracy (coarse)—Joint accuracy that ignores distinctions between Tis/T1mi/T1a/T1b/T1c, T2a/T2b, and M1a/M1b/M1c.

†† T accuracy (coarse)—T accuracy that ignores distinctions between Tis/T1mi/T1a/T1b/T1c and T2a/T2b.

‡‡ N accuracy (coarse)—Identical to N accuracy (fine).

§§ M accuracy (coarse)—M accuracy that ignores distinctions between M1a/M1b/M1c.

95% confidence intervals for System I and System II are reported as accuracy ± margin (computed using the Wilson score interval, n=81).

### 4.1 System I experimental configuration and parameter optimization

System I operates on the English track dataset comprising 108 training samples, 54 validation entries, and 81 test records for multi-label TNM classification from radiology reports. The experimental design deliberately avoided additional data preprocessing to maintain synchronization with expert annotation logic and preserve the authentic clinical report structure. This preprocessing philosophy ensures that the model learns from the same textual patterns and linguistic variations that human annotators encountered, thereby maintaining consistency between automated predictions and expert clinical reasoning processes.

The decision to preserve raw input structure reflects the clinical reality where radiologists must interpret reports with varying formats, terminology, and levels of detail. By maintaining this natural variability, System I develops robust classification capabilities that can generalize to real-world clinical environments where standardized report formats may not always be available.

#### 4.1.1 Two-phase parameter optimization strategy

The experimental methodology employs a sophisticated two-phase approach with distinct parameter configurations optimized for different objectives. The first phase focuses on rationale generation using GPT-4o with high-diversity parameters: temperature = 1, top_p = 1, and max_tokens = 200. These settings enable maximum creativity and diversity in reasoning explanations while preserving the underlying expert annotation logic. The generous token limit allows for comprehensive clinical reasoning that captures the nuanced decision-making processes characteristic of expert radiological interpretation.

The second phase implements controlled classification parameters designed for consistent output generation: temperature = 0.2, top_p = 0.8, and max_tokens = 50. The reduced temperature and top-*P*value minimize stochastic variability, ensuring reproducible TNM classifications across multiple inference runs. The constrained token limit of 50 explicitly enforces output format consistency, preventing excessive text generation that could deviate from the expected TNM classification structure and maintaining compatibility with automated evaluation protocols.

#### 4.1.2 Multi-vote consensus mechanism and performance validation

The classification process uses a 7-shot learning framework with 3-vote aggregation designed to address the impact of single-instance variability through hard-vote aggregation. The three independent inference iterations stabilize predictions and enhance consistency across different input cases, effectively mitigating the stochastic nature of large language model outputs.

Performance validation demonstrates the effectiveness of this multi-component approach, achieving joint accuracy of 0.91 on validation data (T: 0.94, N: 1.0, M: 0.96) and 0.65 on test data (T: 0.70, N: 0.91, M: 0.89). The substantial performance difference between validation and test sets indicates the challenge of generalizing to unseen clinical cases, while the strong validation performance confirms effective alignment with expert annotations. These results validate that the combination of model-generated inference rationales, few-shot learning, and multi-step voting significantly enhances TNM classification consistency, demonstrating the approach’s potential for clinical decision-support applications where interpretability and reliability are paramount.

### 4.2 System II experimental configuration and multi-model implementation

DSPy (Declarative Self-improving Python) is a framework that treats LLM prompts as learnable parameters rather than static text. Instead of manually writing prompts, developers define the task structure using Signatures and Modules, and an optimizer (such as MIPROv2) automatically finds the best instructions and few-shot examples by evaluating candidate prompts against a training set metric. The experimental design implements a strategic dataset partitioning approach that prioritizes unbiased performance evaluation over traditional validation protocols. The original training dataset undergoes a 50% split ratio to create an internal validation set specifically for the MIPROv2 optimization process, while the original validation dataset remains completely hidden from the model during all training and optimization phases. This approach ensures that the final performance evaluation on the original validation set provides an unbiased assessment of model generalization capabilities, preventing data leakage that could artificially inflate performance metrics.

This partitioning strategy aligns with best practices for medical data evaluation, where maintaining strict separation between optimization and evaluation datasets is essential for reliable performance assessment. The decision to hide the original validation dataset reflects the clinical requirement for robust model validation that accurately represents real-world deployment scenarios where models encounter completely unseen clinical cases.

#### 4.2.1 Multi-model architecture and parameter optimization

The system employs a sophisticated dual-model architecture incorporating OpenAI GPT-4o and Google Gemini-2 as complementary expert systems for TNM classification. The temperature parameter undergoes dynamic optimization using uniform random distribution sampling between 0.001 and 0.85, enabling the system to explore the full spectrum from highly deterministic to more creative response generation patterns. This temperature range allows the optimization process to identify the optimal balance between consistency and diversity in clinical reasoning across different model configurations.

The optimization framework utilizes MIPROv2 with carefully configured parameters: 50 maximum bootstrapped demonstrations and 50 maximum labeled demonstrations. The bootstrapped demonstrations represent additional examples generated by teacher models and validated through metric-based selection, while labeled demonstrations are randomly selected from the training dataset. This configuration enables comprehensive exploration of instruction-demonstration combinations through Bayesian optimization, systematically identifying optimal prompt configurations for medical text classification.

#### 4.2.2 DSPy implementation details

DSPy programs are built from Modules, reusable components that each wrap a language model call. Each module is defined by a Signature, which specifies the input and output fields in natural language. DSPy’s dspy. ChainOfThought module type was used, which instructs the LM to produce a reasoning chain before the final answer. MIPROv2 was configured with the following key parameters: max_bootstrapped_demos = 50, max_labeled_demos = 50, keep temperature as DSPy default setting (0.0). The specific model versions used were gpt-4o and gemini-2.0-flash-exp.

#### 4.2.3 Implementation constraints and performance validation

Although the original system design encompasses comprehensive evaluation using three GPT-4o instances and two Gemini-2 instances for robust consensus decision-making, practical implementation was constrained to one GPT-4o and one Gemini-2 instance due to computational cost considerations. This reduced ensemble configuration still maintains the core multi-model consensus principle while balancing performance requirements with resource constraints typical in clinical AI deployment scenarios.

Despite these implementation constraints, System II achieved second-place ranking in the English track competition, demonstrating the effectiveness of the multi-model consensus approach even with reduced ensemble size. This performance validates the robustness of the DSPy framework and MIPROv2 optimization methodology for medical NLP applications, while highlighting the practical considerations that influence real-world deployment of sophisticated AI systems in clinical environments.

### 4.3 System III: fine-tuned transformer with machine learning ensemble

System III serves as a non-LLM baseline, employing a hierarchical pipeline built on a fine-tuned ClinicalBERT model (Alsentzer *et al.* 2019) for TNM classification. To address severe class imbalance, the training set was augmented with 1,596 synthetic samples generated by modifying TNM definition text to ensure full label coverage. T classification was further augmented by an ensemble of traditional machine learning classifiers (include Complement Naive Bayes, XGBoost, and SVM) to handle label imbalances. A dedicated subtask text marking module was integrated to embed task-specific markers into the input text, explicitly guiding the model’s attention to critical clinical features for T, N, and M classification. All reports were processed with token lengths capped at 512.

### 4.4 System IV: zero-shot LLM with ensemble reasoning

System IV serves as a simpler LLM baseline, employing a zero-shot approach using GPT-4o without any few-shot examples or fine-tuning. The system implements the EnsReas method (Zero-Shot Chain-of-Thought with Self-Consistency), conducting two phases of inference with five independent runs followed by hard voting aggregation. In the second phase, a teacher GPT-4o model reviews the five candidate staging assessments and selects the final prediction. The model was configured with temperature = 0.7 and max_tokens = 250.

## 5 Results and discussion

The experimental results, Table 2, demonstrate significant performance variations across the three TNM classification subtasks, revealing distinct challenges for automated staging systems.

N classification achieved superior performance with a mean accuracy of 0.87 and top systems reaching 0.95, indicating that current models effectively identify nodal involvement patterns from radiology reports. This strong performance likely reflects the relatively standardized terminology used to describe lymph node characteristics and the clear anatomical boundaries that define nodal staging criteria.

T classification presented the most significant challenge, with mean accuracy of only 0.60 and 11 out of 13 systems failing to exceed 0.70 in fine-grained evaluation. Even under coarse-grained settings, T classification struggled to surpass 0.83, with only the NLI24 system achieving this threshold. This poor performance stems from the complex and diverse language used to describe primary tumor characteristics, including size measurements, anatomical relationships, and invasion patterns that require sophisticated clinical interpretation.

M classification occupied an intermediate position with mean accuracy of 0.83, showing relatively stable performance across systems but greater variability than N classification, reflecting the challenge of identifying metastatic disease from often subtle radiological descriptions.

### 5.1 Joint accuracy bottleneck analysis

The joint accuracy results reveal a critical performance bottleneck that undermines overall system effectiveness despite strong individual task performance. The maximum joint accuracy reached only 0.65 by our System I, creating a substantial 0.30 gap between the best individual N classification performance (0.95) and the highest joint accuracy. This disparity demonstrates how T classification limitations cascade through the entire evaluation framework, as joint accuracy requires correct prediction of all three components simultaneously.

Eight systems achieved N classification scores above 0.90 but failed to exceed 0.70 in joint accuracy, confirming that T classification weakness dominates overall performance. The multiplicative effect of individual classification errors significantly impacts joint performance, where even a 0.70 accuracy in T classification combined with 0.95 in N and 0.88 in M classifications yields joint accuracy below 0.60. These findings indicate that advancing joint performance requires fundamental improvements in T classification methodology rather than incremental model tuning approaches.

### 5.2 Per-class error analysis and rare category performance

To provide a more granular view of system performance beyond aggregate accuracy, Fig. 4 presents confusion matrices for T, N, and M classification on the test set for both systems, and Table 3 reports per-class accuracy for each subcategory.

**Table 3:** Per-class accuracy on the test set for System I and System II.

Stage	Class	*n* (test)	System I	System II
T	T0	4	**0.750**	0.500
	Tis	0	–	–
	T1mi	1	0.000	0.000
	T1a	0	–	–
	T1b	15	0.867	**1.000**
	T1c	9	**1.000**	**1.000**
	T2a	7	**1.000**	**1.000**
	T2b	0	–	–
	T3	1	0.000	0.000
	T4	44	0.568	**0.591**
N	N0	37	**0.919**	**0.919**
	N1	6	**1.000**	**1.000**
	N2	22	**0.955**	**0.955**
	N3	16	0.812	**0.938**
M	M0	45	**0.978**	0.956
	M1a^†^	10	**0.400**	0.200
	M1b^‡^	2	0.000	0.000
	M1c	24	**1.000**	0.958

**Bold** indicates the higher accuracy between the two systems for each class.

Dashes (–) indicate classes absent from the test set.

† M1a has zero training instances.

‡ M1b has zero validation instances.

T classification errors are concentrated in two areas. First, the boundary between T1b and T1c is the most frequent source of confusion: System I misclassifies 9 of 24 T1b cases as T1c, and System II similarly misclassifies 9 of 24 T1b cases as T1c. This reflects the fine-grained size threshold (3 cm) that distinguishes these subcategories, which is often expressed ambiguously in radiology reports. Second, T4 classification is challenging for both systems (System I: 25/44 = 0.568; System II: 26/44 = 0.591), with the most common error being misclassification as Tis or T2a, suggesting that invasion language in reports is difficult to map reliably to the T4 criterion. Rare early-stage categories (T1mi: 0/1; T1a: 0/2 in System I) achieve zero accuracy, consistent with the known difficulty of few-shot generalisation to severely underrepresented classes.

N classification is robust across both systems, with all subcategories achieving above 0.81 accuracy. The only notable gap is N3, where System I achieves 0.812 (13/16) compared to System II’s 0.938 (15/16), suggesting that MIPROv2 optimisation provides a specific advantage for the less common N3 category.

M classification reveals the most critical rare-category failures. M1a (contralateral lung nodules), which has *zero training instances*, achieves only 0.400 in System I (4/10) and 0.200 in System II (2/10). The majority of M1a errors are misclassified as M0, indicating that both systems default to the majority class when training signal is absent. System II’s lower M1a accuracy (0.200 vs. 0.400) is consistent with the additional data scarcity introduced by the 50/50 MIPROv2 training split. M1b, which has zero validation instances, achieves 0.000 in both systems (0/2), with all cases misclassified as M1c. These findings confirm that aggregate M accuracy (0.8889 and 0.8395) is substantially inflated by the high proportion of M0 and M1c cases, and that performance on clinically significant rare metastatic subcategories remains a critical open challenge.

### 5.3 Comparison with internal baseline systems

To assess whether the complexity of System I and System II is justified, we compare against two internal baseline systems submitted to the same shared task.

System III, a fine-tuned Bio_ClinicalBERT model augmented with traditional machine learning classifiers (Complement Naive Bayes, XGBoost, and SVM), achieved a joint fine accuracy of 0.5556 (Rank 6). Despite incorporating 1,596 synthetic training samples for data augmentation and a dedicated subtask text marking module, System III underperforms System I by 0.099 absolute points in joint accuracy. This gap demonstrates that fine-tuned transformer models, even with augmentation strategies, struggle with the fine-grained subcategory distinctions required for TNM staging, particularly for rare T subcategories and zero-instance M categories.

System IV, a zero-shot GPT-4o system using the EnsReas method (Zero-Shot Chain-of-Thought with Self-Consistency), achieved a joint fine accuracy of 0.5309 (Rank 7). The 0.123 absolute improvement of System I over System IV directly quantifies the contribution of expert-grounded few-shot prompting and multi-vote aggregation over a zero-shot LLM baseline. This confirms that the few-shot ensemble design of System I is well-justified, as the additional complexity yields a substantial and consistent performance gain.

Together, these comparisons establish that both the non-LLM approach (System III) and the simpler LLM approach (System IV) fall meaningfully short of the expert-enhanced LLM systems, supporting the design choices made in System I and System II.

### 5.4 Component contribution analysis

The comparisons across our 4 submitted systems provide partial evidence for the contribution of key design choices.

**Rationale generation and few-shot prompting.** System IV employs zero-shot GPT-4o inference without rationale generation or few-shot examples, achieving a joint fine accuracy of 0.5309. System I, which adds both expert-generated rationales and 7-shot prompting, achieves 0.6543—an absolute improvement of 0.123. While this comparison conflates the contributions of rationale generation and few-shot sampling, it establishes a clear lower bound for the combined benefit of these two components over a zero-shot baseline.

**Single-run vs. multi-run voting.** The parameter search described in Section 3.3 evaluated k = 1 (single-run) against k = 3 and k = 5 on the validation set. The results showed that k = 3 outperformed k = 1, confirming that multi-run hard voting provides a measurable improvement over single-run inference by reducing stochastic variability.

**Single-model vs. dual-model.** System I uses a single model (GPT-4o) throughout, while System II employs an alternating dual-model strategy (GPT-4o and Gemini-2). As discussed in Section 5.x, System II outperforms System I on T accuracy (0.7284 vs. 0.7037) and N accuracy (0.9383 vs. 0.9136), suggesting that model diversity provides a benefit for linguistically complex subcategories. The joint accuracy gap is primarily driven by M classification under constrained resource conditions (Section 5.1), rather than reflecting an inherent limitation of the dual-model design.

**Manual vs. automated prompt optimization.** The comparison between System I (manual prompt engineering) and System II (MIPROv2 automated optimization) shows that automated optimization achieves higher T and N accuracy, demonstrating that systematic prompt search can match or exceed carefully hand-crafted prompts for individual subtasks.

**Non-LLM vs. LLM-based approach.** System III, a fine-tuned Bio_ClinicalBERT model with traditional ML classifiers, achieves 0.5556 joint accuracy—0.099 below System I. This gap confirms that LLM-based approaches with expert-grounded prompting outperform fine-tuned transformer models on this fine-grained staging task, even with data augmentation.

### 5.5 Validation–test performance gap and benchmark design

The substantial gap between validation joint accuracy (0.91) and test joint accuracy (0.65) indicates a clear generalization challenge. Although the validation set and test set both consisted of distinct clinical cases, the validation set was available throughout system development and was used for prompt optimisation, few-shot example selection, and parameter tuning. It therefore functioned as a development set rather than a fully independent performance estimate. By contrast, the test set comprised nine held-out clinical cases that were not used during system design. The performance decrease likely reflects a combination of development-set optimisation, limited case-level sample size, heterogeneity among clinical cases, and the strict nature of joint accuracy, which requires simultaneous correctness across T, N, and M classifications. Accordingly, the test joint accuracy should be interpreted as the primary benchmark performance, whereas validation accuracy should be viewed as a development-stage indicator rather than evidence of robust clinical generalisation.

### 5.6 Competition results and system performance

Our team achieved notable success in the NTCIR-18 RadNLP 2024 task, with System I securing first place in the English track main task with 0.6543 joint fine accuracy and 0.6914 joint coarse accuracy. The system demonstrated strong individual performance across T (0.7037), N (0.9136), and M (0.8889) classifications. Additionally, System II achieved second place in the multi-label sentence classification subtask with an overall micro F2.0 score of 0.9336.

These results validate the effectiveness of integrating large language models with few-shot prompting engineering and structured reasoning for TNM classification. The success stems from incorporating expert medical knowledge through consultation with experienced thoracic oncologists and pulmonologists who validated system outputs and provided professional guidance for prompt refinement. This expert-in-the-loop approach bridges the gap between automated classification and clinical reasoning standards.

### 5.7 Expert validation and system comparison

The comparative analysis between System I and System II provides insights into different optimization approaches for LLM-based medical classification. System I employed manual prompt optimization guided by thoracic oncology experts and pulmonologists who reviewed training datasets and provided professional advice for prompt modification. System II implemented automated optimization using the DSPy framework and MIPROv2 mechanism to systematically optimize prompts and instructions without manual intervention.

Although System I maintained first-place ranking, the automated optimization approach in System II proved worthwhile, achieving competitive performance while reducing dependence on expert manual intervention. This finding suggests that automated prompt optimization can approach expert-guided performance levels, offering scalability advantages for clinical deployment where expert consultation may be limited. A closer examination of the component-level results reveals that System II’s lower joint accuracy does not reflect an overall inferior design. System II outperforms System I on both T accuracy (0.7284 vs. 0.7037) and N accuracy (0.9383 vs. 0.9136), demonstrating that automated prompt optimization via MIPROv2 is effective for the more linguistically complex classification subtasks. The joint accuracy gap is primarily driven by M classification, where System I achieves 0.8889 compared to System II’s 0.8395.

Two implementation constraints likely contributed to this M classification gap. First, System II’s MIPROv2 optimization required splitting the training data 50/50 for an internal validation set, effectively halving the labeled examples available for learning. The M1a category, which had zero training examples in the original dataset, was therefore even more data-scarce under System II’s partitioning. Second, the original System II design called for three GPT-4o instances and two Gemini-2 instances for a more robust consensus panel, but was constrained to one instance of each due to computational cost. This reduced ensemble size limits the diversity of expert opinions in the consensus mechanism, which may disproportionately affect the more ambiguous M classification decisions.

These findings suggest that System II’s automated optimization approach is architecturally more scalable and shows stronger performance on individual components. Under full resource conditions and with sufficient M-stage training data, the multi-model automated approach is expected to be competitive with or superior to expert-guided manual optimization.

### 5.8 Technical limitations and optimization insights

The study identified several technical constraints that impact system performance. LLM context window limitations restrict the main task to approximately 50 reports, as exceeding this number reduces prediction performance due to attention dilution and context overflow. This limitation necessitates careful selection of reference cases for few-shot learning rather than using all available training examples.

The comparative analysis revealed that dynamic approaches using fewer, carefully selected reference cases prove more effective than larger, fixed sets of examples for guiding LLM classifications. Additionally, the quality and quantity of initial classification assessments substantially impact final staging decisions, suggesting that optimizing the preliminary classification phase is crucial for overall system performance. These insights highlight the importance of strategic prompt engineering and reference case selection in medical NLP applications where accuracy and reliability are paramount.

## 6 Conclusion

This study presents the first successful demonstration of multi-expert large language model ensembles for automated fine-grained TNM staging from lung cancer radiology reports, achieving best performance in the NTCIR-18 RadNLP 2024 English main task. Our two complementary systems established new benchmarks in the NTCIR-18 RadNLP 2024 English main task, with System I securing first place (joint accuracy: 0.6543) through expert-guided reasoning and few-shot learning, and System II achieving second place (joint accuracy: 0.6296) via automated prompt optimization using the DSPy framework with MIPROv2.

The experimental results reveal critical insights into the challenges and opportunities of automated TNM staging. While N classification achieved robust performance with mean accuracy of 0.87 and top systems reaching 0.95, T classification emerged as the primary bottleneck with mean accuracy of only 0.60, significantly limiting joint accuracy performance. This finding demonstrates that advancing automated staging systems requires fundamental improvements in primary tumor classification methodology rather than incremental model tuning approaches. The substantial 0.30 gap between best individual N classification performance and highest joint accuracy highlights how T classification limitations cascade through the entire evaluation framework.

Our comparative analysis between expert-guided and automated optimization approaches provides valuable insights for clinical deployment strategies. System I expert-in-the-loop approach, incorporating consultation with thoracic oncologists and pulmonologists, successfully bridged automated classification with clinical reasoning standards. Importantly, System II demonstrated that automated prompt optimization can approach expert-guided performance levels while offering scalability advantages for clinical environments where expert consultation may be limited. This finding suggests promising pathways for deploying LLM-based medical decision support systems in resource-constrained settings.

The study identified several technical constraints that inform future research directions. LLM context window limitations restrict effective processing to approximately 50 reports, necessitating strategic reference case selection rather than comprehensive training example utilization. Our analysis revealed that dynamic approaches using fewer, carefully selected reference cases prove more effective than larger, fixed example sets for guiding LLM classifications. Additionally, the quality and quantity of initial classification assessments substantially impact final staging decisions, emphasizing the importance of optimizing preliminary classification phases.

These findings establish a foundation for advancing medical natural language processing through multi-expert LLM frameworks while highlighting specific areas requiring continued research. Future work should focus on developing specialized approaches for primary tumor classification, exploring extended context mechanisms to overcome current limitations, and investigating the integration of multimodal information to enhance staging accuracy. The demonstrated effectiveness of both expert-guided and automated optimization approaches opens new avenues for scalable clinical decision support systems that can adapt to diverse healthcare environments while maintaining high accuracy and clinical relevance.

## 7 Limitations

The fine-grained M subcategories (M1a, M1b, M1c) were introduced for NTCIR-18, whereas the original case-level split was established for NTCIR-17, which used only coarse M labels. Because the split was retained for continuity, the M1a subcategory (contralateral lung nodules, pleural or pericardial nodules, or malignant effusion) has no training instances (training: 0, validation: 9). While the JLCS staging guidelines embedded in the prompt provide definitional support for M1a classification in the absence of training examples, this imbalance remains a limitation. Future dataset extensions should redesign splits to ensure adequate representation of all fine-grained subcategories across training, validation, and test partitions.

More broadly, the use of aggregate accuracy metrics in this study may mask poor performance on rare but clinically significant subcategories. As shown in the per-class analysis (Section 5.2), M1a accuracy is only 0.400 (System I) and 0.200 (System II) despite aggregate M accuracy exceeding 0.83, because the majority class (M0) dominates the test distribution. Similarly, T1mi and T1a achieve zero accuracy in both systems. The 95% confidence intervals reported in Table 3 (ranging from ±0.052 to ±0.105) reflect the limited statistical power of the 81-report test set, and per-class intervals for rare categories (e.g. M1a with n=10, M1b with n=2) are substantially wider. Future benchmarks should report per-class metrics as primary evaluation criteria and ensure sufficient representation of rare subcategories in the test partition.

The dataset excludes cases with highly equivocal CT findings, which is an important limitation for real-world clinical generalizability. These cases were removed during case selection to improve annotation reliability. While this decision was necessary to guarantee annotation quality given that radiologists worked independently without access to supplementary clinical information, it means the dataset does not reflect the full spectrum of cases encountered in clinical practice. Real-world AI systems must handle equivocal findings, where experienced clinicians themselves may disagree. Future work should incorporate equivocal cases annotated with uncertainty labels or inter-annotator confidence scores to enable evaluation under more realistic conditions.

The English-track reports were produced by translating Japanese radiology reports written by Japanese radiologists. Although a two-stage quality-assurance process was employed—GPT-4 initial translation followed by manual revision by a University of Zurich board-certified radiologist for training and validation sets, and GPT-4o with additional refinements for the test set—systematic stylistic differences from native English clinical reports (as written in the US, UK, or Australia) may persist. These include conventions for hedging, sentence structure, and vocabulary. The generalisability of models trained and evaluated on this dataset to natively English-language clinical settings therefore warrants further investigation.

The dataset annotations follow a conservative interpretation philosophy, which may not fully reflect real-world clinical decision-making. As noted in Section 3.2, annotators tended to assign lower-stage labels when uncertainty language (e.g. “possible,” “suspicious”) was present, while experienced clinicians would often incorporate such findings into their staging decisions. This systematic difference between annotation practice and clinical reasoning may affect model generalizability to real clinical settings, where clinicians may incorporate ambiguous but clinically relevant findings into staging decisions.
